# That’s a Wrap! Molecular Drivers Governing Neuronal Nogo Receptor-Dependent Myelin Plasticity and Integrity

**DOI:** 10.3389/fncel.2020.00227

**Published:** 2020-08-04

**Authors:** Steven Petratos, Paschalis Theotokis, Min Jung Kim, Michael F. Azari, Jae Young Lee

**Affiliations:** ^1^Department of Neuroscience, Central Clinical School, Monash University, Prahran, VIC, Australia; ^2^Laboratory of Experimental Neurology and Neuroimmunology, Department of Neurology, AHEPA University Hospital, Thessaloniki, Greece; ^3^ToolGen Inc., Gasan Digital-Ro, Seoul, South Korea

**Keywords:** nogo receptor, Nogo-A, Caspr, paranode, myelin, PrPc, reelin

## Abstract

Myelin is a dynamic membrane that is important for coordinating the fast propagation of action potentials along small or large caliber axons (0.1–10 μm) some of which extend the entire length of the spinal cord. Due to the heterogeneity of electrical and energy demands of the variable neuronal populations, the axo-myelinic and axo-glial interactions that regulate the biophysical properties of myelinated axons also vary in terms of molecular interactions at the membrane interfaces. An important topic of debate in neuroscience is how myelin is maintained and modified under neuronal control and how disruption of this control (due to disease or injury) can initiate and/or propagate neurodegeneration. One of the key molecular signaling cascades that have been investigated in the context of neural injury over the past two decades involves the myelin-associated inhibitory factors (MAIFs) that interact with Nogo receptor 1 (NgR1). Chief among the MAIF superfamily of molecules is a reticulon family protein, Nogo-A, that is established as a potent inhibitor of neurite sprouting and axon regeneration. However, an understated role for NgR1 is its ability to control axo-myelin interactions and Nogo-A specific ligand binding. These interactions may occur at axo-dendritic and axo-glial synapses regulating their functional and dynamic membrane domains. The current review provides a comprehensive analysis of how neuronal NgR1 can regulate myelin thickness and plasticity under normal and disease conditions. Specifically, we discuss how NgR1 plays an important role in regulating paranodal and juxtaparanodal domains through specific signal transduction cascades that are important for microdomain molecular architecture and action potential propagation. Potential therapeutics designed to target NgR1-dependent signaling during disease are being developed in animal models since interference with the involvement of the receptor may facilitate neurological recovery. Hence, the regulatory role played by NgR1 in the axo-myelinic interface is an important research field of clinical significance that requires comprehensive investigation.

## Introduction

Central nervous system (CNS) myelination is a developmentally regulated process governed by key molecular events that integrate dynamic changes at axonal and oligodendroglial cell membranes. Myelination ensures efficient propagation of action potentials along axons. CNS myelin-forming oligodendrocytes initially contact axons that they subsequently may ensheath depending on their electrical activity (Foster et al., 2019). Myelinated axons of the adult CNS demonstrate a substantial degree of plasticity at the axo-glial and axo-myelinic membrane contacts, that are now known to be dynamically modified according to patterns of neural activity (Mitew et al., 2018; Hughes and Appel, 2019). These specific axo-myelinic contacts are coordinated by the myelin-associated glycoprotein (MAG) in cooperation with contactin 1 and Caspr paranodal adhesion molecules to structure the integral myelin domains (Djannatian et al., 2019). Modifications of axonal, and oligodendroglial membranes are regulated by these integral adhesion molecules, being arranged according to structural domains for appropriate morphometry, that are required for axonal propagation through saltatory conduction. A new hypothesis that may shed light on the molecular organization of the structural subdomains of the CNS axo-myelinic unit, is derived from evidence that the Reticulon 4 receptor (RTN4R), known as the Nogo-66 receptor (NgR1), can modify the integral paranodal protein, Caspr, preventing its cleavage and turnover (Lee et al., 2017). This molecular inhibition of Caspr cleavage may ensure the tight segregation of key voltage-gated ion channels, preventing their lateral diffusion from the node of Ranvier and the juxtaparanode through the barrier established by the septate-like junctions at the paranodal domains.

**Figure 1:**
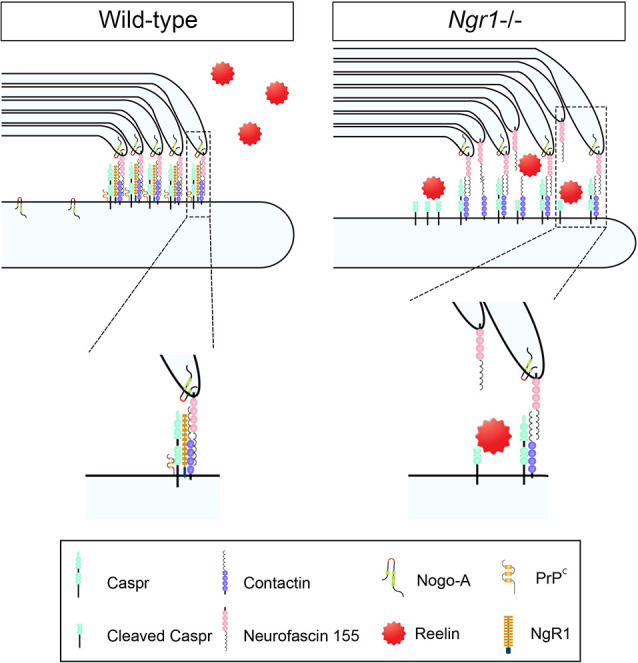
Axo-glial paranodal junctions and a potential regulatory role for Nogo receptor 1 (NgR1): evidence of a disrupted paranode in *ngr1^−/−^* mice. Schematic representation of the proposed unstructured paranodal septate junction in the central nervous system (CNS) myelinated fibers of *ngr1*^−/−^ mice. The absence of NgR1 expression in neurons limits the capacity for cellular prion protein (PrPc) and contactin associated protein (Caspr) to interact, leaving Caspr as a substrate for intramembranous cleavage by Reelin. Expedited Caspr cleavage may promote the decompaction of myelin form the paranodal junctions altering the electrophysiological signature and potentially triggering the continuous turnover of myelin (adapted from Coman et al., 2006).

During disease and trauma, NgR1 has been reported to be upregulated in neurons that exhibit axonal transection or are undergoing degeneration, thereby governing neurite outgrowth inhibition in an extracellular milieu rich in myelin-associated inhibitory factors (MAIFs) or astroglial-derived chondroitin sulfate proteoglycans (CSPGs; for review see Petratos et al., 2010; Lee and Petratos, 2013). During neuroinflammation, NgR1 is also increased in neurons that may potentiate axonal degeneration through downstream signaling that can destabilize or disassemble the axonal cytoskeleton following Nogo-A-dependent ligation (Petratos et al., 2012; Lee et al., 2019). Of functional importance, is that during the development of the CNS visual system, NgR1 is strongly expressed in parvalbumin-positive interneurons to restrict the critical period of ocular dominance plasticity based on a disinhibitory microcircuit (Stephany et al., 2016). This raises the possibility that the expression of NgR1 throughout the neuronal soma, dendrites, and axons can be variable and inducible to control activity-dependent plasticity that may include axo-glial synapse-like structures. With recent evidence implicating that NgR1 can coordinate plasticity and memory formation in specific cortical regions (Karlsson et al., 2016) and the evidence for myelin plasticity to be center stage of human learning and cognition (Sampaio-Baptista et al., 2020), the investigative path to NgR1-dependent myelin plasticity (see Figure 1) is an integral open question in neurobiology that requires elucidation due to the numerous therapeutic strategies being developed for individuals with mainly acquired neurological diseases.

**Figure 2:**
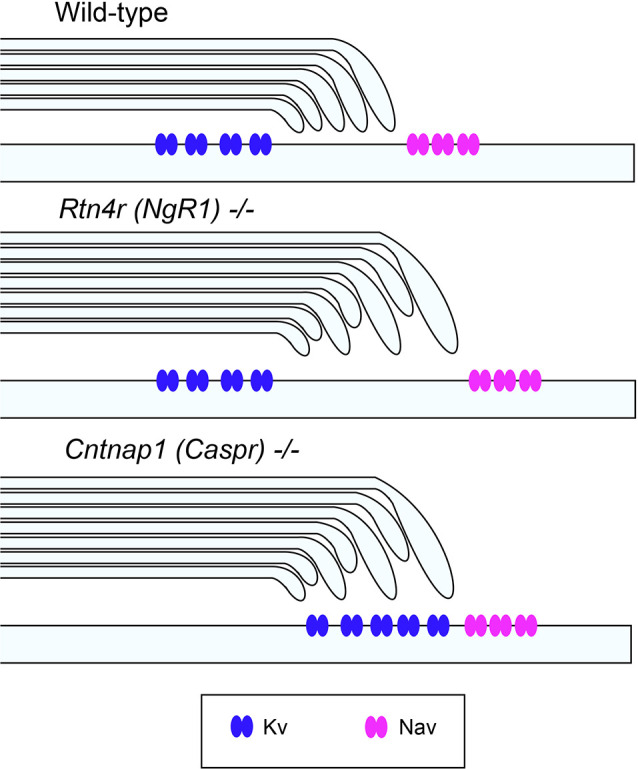
Unaltered ion channel distribution in the axo-myelinic junction of the CNS of *ngr1^−/−^* mice. Schematic representation of the proposed ion channel distribution in the CNS myelinated fibers of wild-type, *ngr1*^−/−^ and *caspr*^−/−^ mice. In *caspr*^−/−^, juxtaparanodal Kv channels are displaced and distributed throughout the paranodal region, whereas in *ngr1*^−/−^, although lengthening of paranodal Caspr was found, ion channel distribution could not be identified (Lee et al., 2017).

These questions can only be addressed when we investigate the molecular dynamics of the axo-glial and axo-myelinic interfaces during development and disease. From an ultrastructural vantage point, key subdomains are present at paranodal regions that flank the nodes of Ranvier, enriched in voltage-gated sodium channels (Caldwell et al., 2000). The establishment of the node is essential for the fast propagation of action potentials by conduction through the internode. Paranodal regions contain high concentrations of proteins such as contactin-1 and contactin associated protein (Caspr), which play an important role in establishing the neuronal membrane anchoring point for the oligodendroglial neurofascin 155 (NF155) protein (Gollan et al., 2003). Mice, deficient in the Caspr gene *(cntnap1)* exhibit abnormal formation of CNS nodes, since Caspr regulates the binding of NF155 to contactin 1 through direct interaction and processing for its membrane localization (Gollan et al., 2003). Genetic knockout models of contactin, Caspr, or neurofascin 155 display a similar pattern of paranodal disorganization (Bhat et al., 2001). This paranodal disruption involves the misdistribution of juxtaparanodal voltage-gated K^+^ channel 1.2 (K_v_1.2) close to and encroaching into the nodal gap (Coman et al., 2006; Howell et al., 2006), thereby implicating a disruption to axonal-myelin membrane integrity (Figure 2). This disruption is manifest as a loss of transverse bands and increased intermediate distance with everted paranodal loops in *cntnap1* (Caspr) mutant mice that although display normal myelination but exhibit disrupted paranodal septate-like junctions, resulting in reduced axonal conduction velocity (CV). Therefore, it is plausible that in the presence of axonal degeneration without prominent demyelination observed in some MS lesions (Bjartmar et al., 2001), such paranodal disruption, leading to axonal degeneration, may precede overt demyelination (Desmaziàres et al., 2012).

Evidence gathered from auditory processing fibers within the brainstem has shown that elevated CVs occur in the large diameter myelinated axons that respond to low-frequency sound waves, with reduced internodal distances (Ford et al., 2015). The morphometric variability in low frequency-respondent auditory processing fibers highlights that there exists a non-canonical array of myelinated segments along axonal fibers to tune axonal conduction in time and space. The investigations that proposed this more rational theory of tunable conduction in the CNS, initially showed that variable diameters and internodal distances can occur along the length of axons to modify action potentials and conduction at anatomically relevant junctures when capacitance modulation can be tuned to regulate synaptic transmission outcomes and plasticity (Ford et al., 2015). These investigators then identified that the conduction speeds generated through computer simulations of the morphometric parameters measured in the mammalian auditory cochlear nucleus globular bush cells (GBC) processing fibers, predicted that action potential propagation speeds are modified to confer the simultaneous arrival times at the giant calyx of Held presynaptic terminal, establishing a precise temporal association of input signals for binaural recognition. These structural variances, therefore, seem integral to how CNS axons can integrate information post-synaptically in disparities of time and space. It would be of great significance if we could identify the molecular drivers that diversify the proximal and distal myelinated segments that filter the current flow since this can occur during demyelinating disease and re-establishment of CNS fiber capacitance may limit the deficits manifest in conditions such as MS (Ortiz et al., 2019).

## Nogo Receptor 1 (Ngr1) Regulates Neuronal Morphology and Synaptic Plasticity

**Figure 3:**
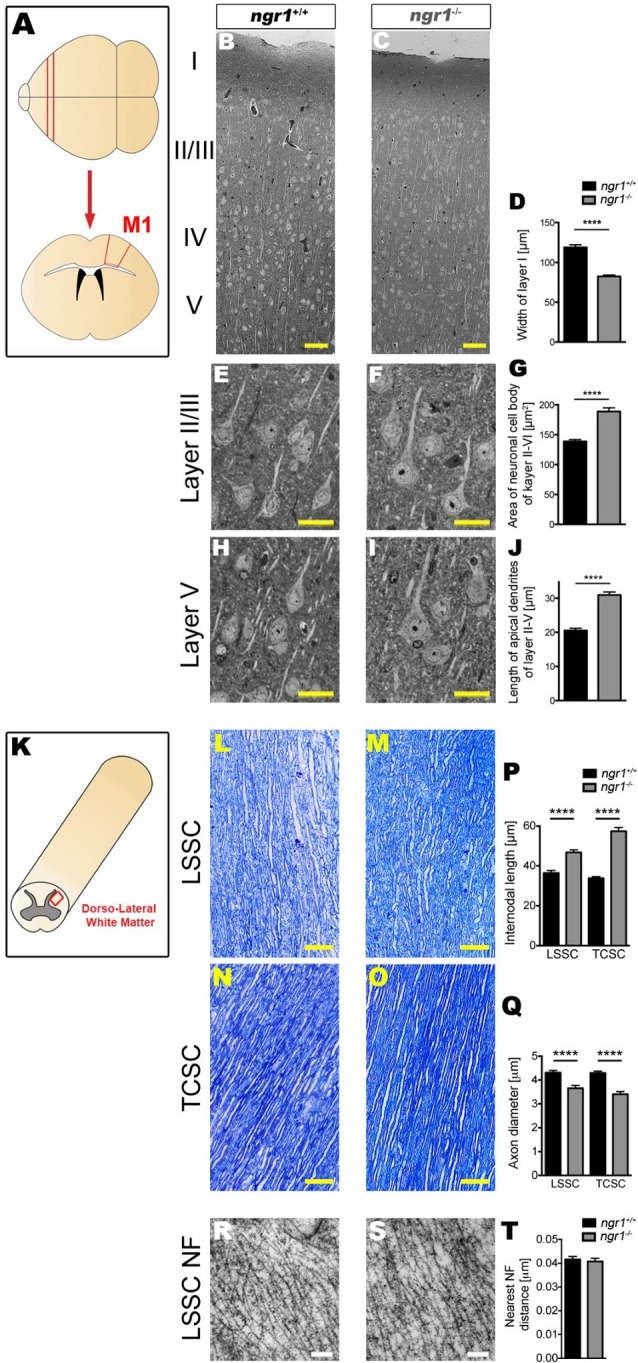
Altered neuronal architecture within the frontal cortex and altered axonal and myelin architecture in the spinal cord of *ngr1^−/−^* mice. For detailed methods please see the Supplementary Information. **(A)** Illustration of the M1 frontal motor cortex region from which samples were imaged. **(B–J)** Semi-thin sections from the M1 regions of *ngr1^+/+^* and *ngr1*^−/−^ mice revealed a different neuronal morphology. **(B,C)** Light microscopic images of the M1 cortical layer from layer I to V in *ngr1^+/+^* and *ngr1*^−/−^ mice (scale bar = 50 μm). **(D)** Measurements of the molecular layer width; layer I, was significantly shorter in *ngr1*^−/−^ compared to *ngr1^+/+^* mice. **(E–I)** Representative high magnification images of pyramidal neurons from **(E,F)** Layer II/III **(H,I)** and layer V. Both **(G)** area and **(J)** length measurements obtained for the apical dendrites of neurons within the cortical layers II-V were found to be increased in *ngr1*^−/−^ compared to *ngr1^+/+^* mice. **(K)** Illustration of the dorsolateral white matter region of the spinal cord from which samples were imaged. Representative image showing how internodal length and axonal diameters measured is shown below. **(K–Q)** Toluidine blue-stained semi-thin (1 μm) dorsolateral white matter sections of lumbosacral (LSSC) and thoracic-cervical (TCSC) spinal cords (red rectangle region) of both adult *ngr1*^+/+^ and *ngr1*^−/−^ mice (scale bars = 50 μm). **(P)** Internodes of *ngr1*^−/−^ were significantly longer in both LSSC and TCSC when compared with *ngr1^+/+^*. **(Q)** Axonal diameters of *ngr1*^−/−^ mice were significantly smaller in both LSSC and TCSC when compared with *ngr1*^+/+^ mice. **(R,S)** Ultra-thin (100 nm) electron micrograph longitudinal sections of LSSC from adult **(R)** *ngr1*^+/+^ and **(S)** *ngr1*^−/−^ mice showing normal ultra-structure of neurofilaments in both genotypes (scale bar = 100 nm). **(T)** Quantification of nearest neighbor distances between neurofilaments in myelinated axons of descending fiber tracts in *ngr1^+/+^* and *ngr1*^−/−^ LSSC (****P < 0.0001, *n* = 8 for both genotypes).

The NgR1 is a high-affinity receptor for the Nogo-66 extracellular C-terminus domain of Nogo-A, an integral oligodendroglial and myelin membrane protein. NgR1 regulates the experience-dependent turnover of dendritic spines and limits synaptic plasticity in the cortical gray matter (Akbik et al., 2013). Such modifications in neuronal axodendritic architecture are dependent on myelin ligands that bind avidly to the leucine-rich repeat region (LRR) of NgR1 (Dickendesher et al., 2012; Akbik et al., 2013). We have recently proposed a novel NgR1-dependent mechanism regulating myelin plasticity governed at paranodal regions of the CNS that is disinhibited in mice lacking the Ngr1 allele (Figure 1). Indeed, our investigations have identified further ultrastructural changes in the prefrontal cortex of *ngr1*^−/−^ mice whereby the cell bodies of projection neurons in the cortical region were enlarged with elongated apical dendrites (Figures 3E–G). This profile was in opposition to the width of cortical Layer I but in-line with that observed in Layers II-V (Figures 3B–D,H–J, respectively). Cortical layer I (molecular layer) in *ngr1*^−/−^ mice, showed a decrease in thickness (Figures 3B–D) suggesting reduced density of myelinated fibers or reduction in the unmyelinated axonal densities residing in this layer. A plausible explanation for this reduced fiber density may be that the dense perineural nets surrounding cortical neurons can exhibit an altered composition of chondroitin sulfate proteoglycans (CSPGs), modifying the connectivity in the cortex of the *ngr1*^−/−^ mice, particularly since NgR1 can be an alternate receptor for CSPGs (Ye and Miao, 2013). However, at first glance, these observations do not align with the concept of modified neuronal architecture among the projection neurons since no such alterations could be detected and neurofilaments were evenly spaced in the cortex of both *ngr1*^−/−^ and *ngr1^+/+^* mice (Figures 3R–T; Lee et al., 2017). Nevertheless, this apparent contradiction can be reconciled when the plasticity of adult cortical neurons of *ngr1*^−/−^ mice is taken into account (Dickendesher et al., 2012). In the M1 and V1 cortex of *ngr1*^−/−^ mice, the gains and losses of dendritic spines are approximately double that of control mice, with potentiated turnover implicating synaptic plasticity (Dickendesher et al., 2012). This suggests that a gate in plasticity is lacking in the *ngr1*^−/−^ mice since the potent Nogo-A neurite outgrowth inhibitor expressed in mature oligodendrocytes in adulthood cannot limit the membrane-dependent dendritic or axonal varicosity formation. Therapeutics are being developed to target NgR1-dependent membrane interactions in various disease paradigms (for review see Petratos et al., 2012; Lee and Petratos, 2013; Lee et al., 2014; Kim et al., 2018). Therefore, it is important to dissect and define the precise mechanisms in which NgR1 can regulate plasticity at the axo-glial synapse.

We know that NgR1 may be important in ocular dominance plasticity within the visual cortex of naïve mice (McGee et al., 2005; Stephany et al., 2014). We also know that acute electrophysiological plasticity can be regulated by Nogo-A-NgR1 signaling (Raiker et al., 2010). Moreover, it has been shown that adult synaptic plasticity and dendritic architecture, can be regulated by NgR1 (Lee et al., 2008; Raiker et al., 2010; Zagrebelsky et al., 2010; Delekate et al., 2011; Wills et al., 2012; Akbik et al., 2013). This regulatory role of NgR1 in synaptic plasticity has been linked to a neuropsychological phenotype that mimics schizophrenia (Budel et al., 2008). Together, these results indicate that NgR1 has a distinct role in the regulation of neural architecture. However, whether there are ultrastructural differences within CNS white matter tracts, related to axo-glial connectivity and dynamics, has not been investigated to date and is a valid line of investigation. This is so, since myelin plasticity is now well documented in the enhancement of cognitive function, an established role in NgR1-dependent physiology.

An alternate hypothesis that may explain the morphometric variability in the cortical architecture observed in our *ngr1^−/−^* mice may involve the level and or dynamics of intracortical myelination, regulating dendritic arborization and hence maturation. It has been demonstrated that the cognate high-affinity ligand for NgR1, Nogo-A, is not only localized to oligodendroglial plasma membranes but has been shown that its neuronal expression to be specifically observed during development and can limit dendritogenesis (Petrinovic et al., 2013). These investigators demonstrated that Nogo-A knockout mice exhibited elaborate Purkinje cell dendritic trees, greater synaptic strength between parallel fiber terminals, and Purkinje cell post-synaptic densities with potentiated excitatory presynaptic current (EPSC). The data suggest neuronal Nogo-A expression limits the development and synaptic strength, at least of the cerebellar cortex (Petrinovic et al., 2013). Moreover, it has been established that both oligodendroglial-specific and neuronal-specific Nogo-A can regulate the dendritic arborization with distal vs. proximal dendrites influenced respectively (Zemmar et al., 2018). However, the stabilization of dendritic synaptic fields demonstrated by Nogo-A in dendrites in the hippocampal cortex cannot be replicated in axons, which is governed by NgR1/Nogo-A suggesting a key signaling mechanism driving axo-dendritic synaptic plasticity (Zagrebelsky et al., 2010). However, whether these fundamental receptor/ligand interactions occur at axo-oligodendroglial synapses that govern the dendritic maturation in simplistic intracortical regions (Glasser et al., 2014), is yet to be elucidated.

## Ngr1 Regulates Myelinated Fiber Structure and Function

**Figure 4:**
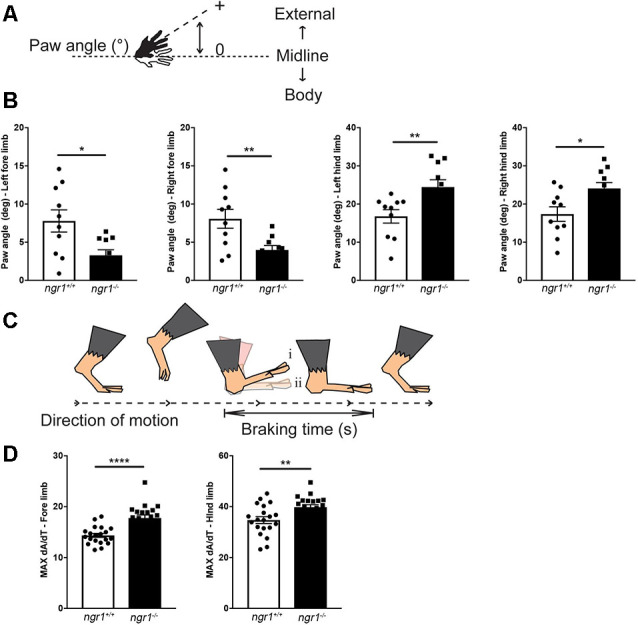
Altered paw angle and rate of deceleration during the braking phase in naïve *ngr1^−/−^* mice. For detailed methods please see the Supplementary Information. **(A)** Absolute paw angle is the angle that the paw makes with the long axis of the direction of motion (midline). Higher absolute paw angles represent greater degrees of external rotation. **(B)** The forepaws of naïve NgR1-KO (*ngr1*^−/−^) mice are less externally rotated than naïve wildtype mice (*ngr1^+/+^*), while NgR1-KO hind paws are more externally rotated than WT mice. **(C)** The maximal rate of change of the paw area (MAX dA/dT) provides a measure of how rapidly the animal decelerates during the braking phase. An increased MAX dA/dT represents a greater change in paw area over the same amount of time; therefore, compared to its littermate (i), the animal is placing its paw down faster (ii; modified from Vincelette et al., 2007). **(D)** KO mice had an increased MAX dA/dT compared to WT mice for both fore- and hind-paws. Data are presented as mean ± SEM. Unpaired *t*-test. *P < 0.05, **P < 0.01, ****P < 0.0001.

We have recently performed systematic ultrastructural analyses of the dorsolateral spinal cord white matter tracts in the thoracic and lumbosacral segments of the *ngr1^−/−^* mice that have revealed longer mean internodal lengths when compared to wildtype littermate controls. Mean axon diameters were also reduced in the *ngr1*^−/−^ compared to the wild type littermate controls (Figure 3). Along with the observed reduced axonal caliber with commensurate thinner myelin exhibited by mice mutant for the *ngr1* allele, we also demonstrated that these mice had increased numbers of thin fibers in spinal cord fascicles (Lee et al., 2017). Indeed, these ultrastructural changes may explain the altered functional and locomotor performance exhibited by these mice (Figure 4).

Our gait analysis of naïve wildtype and *ngr1^−/−^* animals has identified altered paw angles and the rate of deceleration during the braking phase of the gait cycle. Forepaws of naïve *ngr1*^−/−^ mice were more internally rotated (narrower absolute paw angles), and their hind paws more externally rotated (wider absolute paw angles) than naïve wild-type mice (Figure 4). These observations suggest *ngr1*^−/−^ mice may have difficulty in balancing themselves during a stance. Another feature of abnormal gait in the *ngr1*^−/−^ mouse is a significant increase in the maximal rate of change of paw area (MAX dA/dT), a measure of how rapidly the animal decelerates during the braking phase. These observations need to be further investigated to elucidate the precise neurophysiological role/s of NgR1 in gait regulation, and further dissect how changes in axon and myelin properties observed in the *ngr1*^−/−^ mice can potentiate such neurobehavioral outcomes.

## Neuronal Ngr1 and the Axo-Myelin Interface

So how can NgR1, a high-affinity pleiotropic receptor that inhibits neurite outgrowth, regulate the plasticity of the axo-glial unit? The dynamics of integral adhesion proteins regulating axo-myelinic membrane interactions are related to the rate of activity in axonal conduction (Mensch et al., 2015; Almeida and Lyons, 2017; Hughes and Appel, 2019; Ortiz et al., 2019; Saifetiarova et al., 2017). A thorough analysis of the cellular and molecular ultrastructure of axo-glial units within the white matter of *ngr1^−/−^* mice shows an altered adhesion of paranodal myelin with the axolemma that corresponds with a preserved expression of Kv1.2 ion channels but distributed (or the diffusion of) contactin-related protein throughout the node and internode regions (Lee et al., 2017). Also, the expression of NgR1 is dependent on neuronal activity and postsynaptic density formation, limiting hippocampal neuron dendritic growth and plasticity (Wills et al., 2012). It is established that neurons of the visual system lacking the expression of NgR1 exhibit increased levels of excitatory synaptic input and plasticity (Stephany et al., 2018). Furthermore, we have recently discovered that isolated cortical neurons from *ngr1*^−/−^ mice exhibit potentiated anterograde vesicular axonal transport when compared to isolated cortical neurons from wild type littermates (Lee et al., 2019). Despite elevated neurotransmission observed in *ngr1*^−/−^ mice, the velocities of compound action potentials (CAPs) are reduced in both spinal cord dorsal white matter tracts and optic nerves of these mice (Lee et al., 2017). Taken together, these data suggest a degree of complexity in electrophysiological mechanisms governed by NgR1 in white matter tracts of the CNS.

We have previously demonstrated NgR1 to be a key regulator of the distribution of an integral paranodal protein, Caspr, along with the intramembranous cleavage of the paranodal protein at the junction (Lee et al., 2017). This finding correlated with the altered ultrastructural organization at the paranode and internode of *ngr1^−/−^* mice and disrupted expression and localization of other key subdomain proteins and ion channels, resulting in delayed conduction velocity. Hence, through extensive ultrastructural molecular and electrophysiological studies of *ngr1*^−/−^ mice, we identified an indirect role in the regulation of axo-glial units for NgR1 (Lee et al., 2017). It is plausible that NgR1 can also regulate the axonal localization of cellular prion protein (PrPc), thereby reducing its interaction with Caspr. Indeed, we found sequestered PrPc within the neuronal somata of the spinal cord gray matter leaving Caspr unbound at the axo-glial junction in *ngr1*^−/−^ mice. Since PrPc has been reported to limit intramembranous Caspr proteolysis through the activity of Reelin at the Laminin-G-like domains (Devanathan et al., 2010), the reduced interaction of PrPc-Caspr may thereby lead to unopposed cleavage of Caspr by Reelin. Indeed, we detected increased proteolytic products of Caspr in the *ngr1*^−/−^ spinal cords, while Reelin levels were sustained. Intriguingly, despite the lack of the *ngr1* allele, Caspr expression was maintained in the context of cleavage (Lee et al., 2017). These data suggest that the *ngr1*^−/−^ adult mouse CNS exhibits immature paranodal junctions and internodal myelin sheaths with constant myelin turnover. These molecular and ultrastructural findings were also electrophysiologically verified by delayed latency in CAP recordings of *ngr1*^−/−^ when compared with wild-type mice. However, these changes in *ngr1*^−/−^ mice did not compromise axonal integrity (Lee et al., 2017). The possibility of developmental myelination being sustained into adulthood in mice that lacking *ngr1* is therefore an open question.

**Figure 5:**
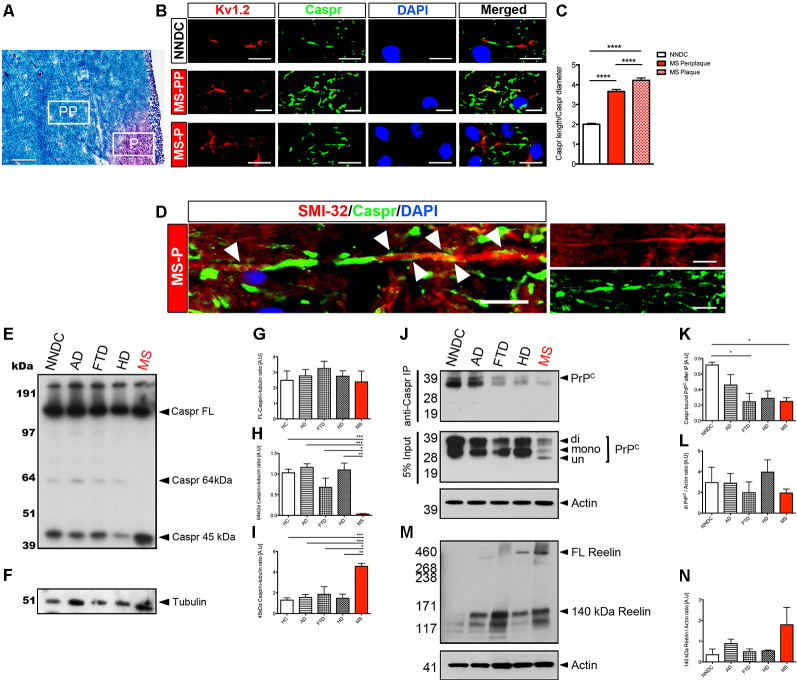
Chronic active MS lesions show Caspr re-distribution, increased NgR1 and axonal damage. For detailed methods please see the Supplementary Information. **(A)** Representative LFB-PAS stained images obtained from progressive MS patient brain tissues. From these, we selected periplaque (PP) and plaque (P) regions to further study Caspr distribution in these areas (scale bar = 500 μm). **(B)** In MS-PP, the co-localization of Caspr with juxtaparanodal Kv1.2 was observed. In MS-P, a significant elongation of Caspr+ segments and disrupted localization of Kv1.2 was observed (scale bar = 10 μm). **(C)** The ratio between the measured length of Caspr+ segments vs. their diameter was significantly increased in MS-PP and MS-P, compared with non-neurological disease control (NNDC) samples. **(D)** Immunostaining of SMI-32, Caspr, and DAPI in serial section showed a re-distribution of Caspr along the whole internode in SMI-32 (+) degenerative axons within the lesion border (white arrowheads indicate diffuse expression of Caspr along the internode; scale bar = 10 μm; one-way ANOVA with *post hoc* Tukey’s test, ****P < 0.0001, *n* = 4 for each patient samples). **(E)** Western blot for Caspr and **(F)** α-tubulin-loading control performed on brain white matter lysates of NNDC, Alzheimer’s disease (AD), frontotemporal dementia (FTD), Huntington’s disease (HD), and MS patients. **(G)** Densitometric quantification of full-length Caspr (FL-Caspr), **(H)** 64kDa degradation product of Caspr and **(I)** 45 kDa Caspr degradation product normalized by α-tubulin loading control (one-way ANOVA *post hoc* Tukey’s test, *P < 0.05, **P < 0.01, ***P < 0.001, *n* = 4 for each patient samples). **(J)** Immunoprecipitation of Caspr and probed with anti-PrP^C^. Western immunoblot for PrP^C^ from 5% input of pre-immunoprecipitation sample shown on the bottom **(K)** Densitometric quantification of Caspr bound PrP^C^ and **(L)** total di-PrP^C^. **(M)** Western blot for Reelin. **(N)** Densitometric quantification of 140 kDa Reelin.

The presence of Caspr at the paranodal junction is a fundamental factor regulating subdomains of the nodes (in coordination with Neurofascins, the nodal and paranodal cytoskeletal scaffolds, the nodal extracellular matrix, along with myelin membrane-bound lipids and glycolipids), that the segregation of sodium and potassium channels that is necessary for the propagation of action potentials at nodes of Ranvier (Bhat et al., 2001; Ohno et al., 2011; Gordon et al., 2014; Laquérriere et al., 2014). Interestingly, the expression of full-length Caspr was maintained in *ngr1^−/−^* mice during EAE indicating that, outside of its well-known role in axonal degeneration, NgR1 may also play a role in neuroinflammation-dependent axoglial dynamics (Lee et al., 2017). This was particularly highlighted when we investigated the chronic active lesions of progressive MS patients (Figure 5). The increased expression of NgR1 that we observed only in MS tissue (Lee et al., 2019) was associated with elevated Reelin-mediated cleavage of Caspr with significant ion channel re-distribution along the axons and potentiated axonal damage (Figure 5). A surprising finding in our study which investigated axo-glial dynamics of *ngr1*^−/−^ mice (Lee et al., 2017), was that although there was increased cleavage of Caspr, no reduction of full-length Caspr was found in the spinal cords of *ngr1*^−/−^ mice, implicating consistent expression and possibly a potential regulation of turnover of myelin by NgR1. We have recently reported that *ngr1*^−/−^ mice also exhibit a sustained expansion of microglia without neuroinflammatory challenge and these cells exhibit increased levels of engulfed myelin proteins (Alrehaili et al., 2018). Furthermore, this observation is consistent with the different expression patterns of Nogo-A found along with the axo-glial units in the spinal cords of *ngr1*^−/−^ mice, as mice deficient in Nogo-A exhibited faster remyelination upon lysolecithin-induced demyelination when compared to wild-type (Chong et al., 2012). Therefore, another open question is whether this endogenous activity, innate to *ngr1*^−/−^ CNS tissues, is a consequence of potentiated clearance of unstructured myelin. This possibility warrants further investigation as it may provide a further understanding of myelin dynamics during disease and possibly also aging.

## Conclusion

How NgR1 regulates the distribution of Caspr in a tightly orchestrated paranodal interaction with its glial membrane proteins that are integral to the synchrony of axonal myelin physiology is an important question. Future research should involve investigations into CNS remyelination in adult *ngr1^−/−^* mice where specific demyelinating lesions are observed during the repair. The cuprizone-mediated experimental demyelination model would be ideal to assess the initiation of paranode and internode formation in the adult CNS, without the influence of neuronal NgR utilizing Cre-deleted NgR1-floxed transgenic mice. Moreover, this model has the added advantage of having no invading autoreactive adaptive peripheral immune cells impacting CNS demyelination/remyelination (as can occur in models such as EAE) and axonopathy that can differentiate the role of NgR1 in myelin turnover during remyelination compared to the axonal degeneration occurring in MOG-induced models. Elucidating the precise coupling of NgR1-dependent neuronal activity with the molecular restructuring of the node of Ranvier and paranodal myelin is a critical line of investigation in neuroscience that will drive the development of future regenerative therapeutic interventions that target the Nogo-A/NgR1 cell signaling mechanism during neurodegenerative diseases governed by the sequelae of inflammation.

## Ethics Statement

The AMREP Animal Ethics Committee (AEC nos. E/1532/2015/M and E/1602/2015/M) reviewed and approved the use of these animals for experimentation in this study, in accordance with the guidelines and regulations set out by the National Health and Medical Research Council of Australia. All animal experiments are governed by the Australian Code for the care and use of animals for scientific purposes (2013) and comply with the Victorian Cruelty to Animals Act 1986. All frozen human deep-cortical white matter tissues used for this study were acquired from the Victorian Brain Bank Network (VBBN) under the National Health and Medical Research Council guidelines and the Monash University Human Research Ethics Committee approval number CF13/1646-2013000831.

## Author Contributions

SP wrote the manuscript. PT, MK, MA and JL edited the manuscript and generated data.

## Conflict of Interest

JL is currently employed by the company ToolGen Inc. The remaining authors declare that the research was conducted in the absence of any commercial or financial relationships that could be construed as a potential conflict of interest.
