# Supplementary material for: That’s a Wrap! Molecular Drivers Governing Neuronal Nogo Receptor-Dependent Myelin Plasticity and Integrity
Source: Front Cell Neurosci. 2020 Aug 4;14:227. doi: 10.3389/fncel.2020.00227 (PMC7417613; doi:10.3389/fncel.2020.00227)
Supplement: Supplementary file 1 [file Data_Sheet_1.docx]

**Supplementary Information**

**Methods**

**Ethics Statement and Animal Experiments:**

The AMREP Animal Ethics Committee (AEC nos. E/1532/2015/M and E/1602/2015/M) approved the use of these animals for experimentation in accordance with the guidelines and regulations set out by the National Health and Medical Research Council of Australia. All animal experiments are governed by the Australian Code for the care and use of animals for scientific purposes (2013) and comply with the Victorian Cruelty to Animals Act 1986.

P120-140 age-matched female naïve *ngr1^+/+^* and *ngr1^-/-^* mice were deeply anesthetized with CO_2_ and perfused with PBS followed by 2.5% glutaraldehyde and 4% paraformaldehyde. The lumbosacral, thoracic-cervical and frontal cortices (M1, primary motor cortex) were dissected and post-fixed in 2.5% glutaraldehyde and 4% paraformaldehyde for 24 hours at 4 °C. Tissues were then washed in 0.1 M cacodylate buffer and contrasted with 1% osmium tetroxide in 0.2 M cacodylate buffer for 2 hours at room temperature. Tissues were then washed in 0.1 M cacodylate buffer and dehydrated in a sequential ethanol gradient from 50-100% and embedded longitudinally in Epon. Semi-thin sections (0.9 μm) were prepared and stained with toluidine-blue and analyzed with an Olympus dotslide BX51 microscope with 20 x objective lens (Olympus). From toluidine-blue stained longitudinal sections of both lumbo-sacral and thoracic-cervival spinal cord, internodal lengths and axonal diameters were measured (At least 500 axons were measured from each mouse, *n* = 8 for both *ngr1*^+/+^ and *ngr1*^-/-^). From toluidine-blue stained coronal sections of frontal cortex, widths of layer I, lengths of apical dendrites and areas of neuronal cell bodies of cortical layer II-V were measured (*n* = 8 for both *ngr1*^+/+^ and *ngr1*^-/-^). Ultra-thin sections (100 nm) were cut and stained with 1% aqueous uranyl acetate. Stained sections were analyzed on a transmission electron microscope (Hitachi H-7500) and captured with a Gatan digital camera (model 791). Nearest neighbor distance was measured (At least 100 axons were measured from each mouse, *n* = 8 for both *ngr1*^+/+^ and *ngr1*^-/-^).

All Data were analyzed using Graph Pad Prism v6.0e. Data represented as mean ± SEM. A two-tailed Student’s t-test was used to determine statistical significance unless otherwise specified.

**Human Ethics Statement**

All frozen human deep-cortical white matter tissues were acquired from the Victorian Brain Bank Network (VBBN) under the National Health and Medical Research Council guidelines and the Monash University Human Research Ethics Committee approval number CF13/1646-2013000831. Tissues included five subjects with non-neurological disease control (NNDC), other neurological disease controls including subjects with Alzheimer’s disease (AD), fronto-temporal dementia (FTD), Huntington’s disease (HD), and progressive MS with active lesions. Post-mortem interval did not exceed 56 h. All specimens were obtained from the frontal lobe deep white matter, frozen under liquid nitrogen and then stored in the Brain Biobank (-80°C until required).
